# Successful Treatment of Lacerations and Fissures of the Os Uteri of Long Standing without Surgical Operation

**Published:** 1885-12-20

**Authors:** Bedford Brown

**Affiliations:** Alexandria, Va.


### SUCCESSFUL TREATMENT OF LACERATIONS AND FISSURES OF THE OS UTERI OF LONG STANDING WITHOUT SURGICAL OPERATION.

**Read before the Medical Society of Virginia, September 17, 1885.**

BY BEDFORD BROWN, M. D., OF ALEXANDRIA, VA.

During the past twelve years the writer has embraced every opportunity to test various means for the purpose of devising some method of treating these injuries successfully, without resort to operations. A very large proportion of females suffering from these afflictions have neither the means nor the opportunity of seeking the aid of the specialist. The writer believes that he has perfected a simple method, which can be used by all practitioners of intelligence, for the treatment of these cases successfully, with time and care, without the necessity of operation. The agents resorted to in this method are entirely of a local character.

Concealed fissures are often found after labor, in the mucous membrane of the cervical canal, and are the cause of an infinite amount of local disease in the form of endocervicitis, hypertrophy of the adjacent tissues, inflammation of the submucous fibrous tissue, leucorrhœa, and often painful menstruation. One of the most usual seats of these fissures is at the internal os. Here the mucous membrane and submucous structure is split through, often to a considerable depth. The rent here, as in the case of fissure of the rectum, will remain as a source of trouble for years.

It is highly probable that these concealed fissures are the prime cause of a very large proportion of the cases of cervicitis, and their consequences, found in women who have borne children. The No. 1 solution of nitrate of silver reaches these wounds effectually, stimulates a new action, and heals them from the bottom readily.

With a view of promoting the healing of the open lacerations and fissures, however small, which act as a centre for the radiation of morbid influence, I have tested every standard agent as a local remedy—Churchill's tincture of iodine, Battey's solution, pure carbolic acid, iodoform, chromic acid, solid nitrate of silver. They all failed signally to promote healing and closure of the laceration. The application of the solid nitrate of silver invariably acted as a violent irritant, increasing inflammation, pain, discharge, and hemorrhage. Finally, a solution of crystals of nitrate of silver, graduated so as to adapt it to the particular parts to be applied and the various stages of progress, was used. The effects of this agent proved that lacerations and fissures of long standing and the most aggravated character, complicated with great degree of inflammation, hypertrophy, subinvolution, and a certain degree of pelvic cellulitis, were curable. An experience in the use of this agent in about twenty cases of lacerations and fissures, from the slightest to the most aggravated form, treated in the past ten years, shows conclusively that the most extensive wounds (many of them bilateral) have been made to heal and close up permanently. The os and cervix have invariably been reduced to their natural appearance and dimensions without contraction.

The treatment requires, for completion, from six to twelve weeks. It can be resorted to regardless of the presence of hypertrophy, inflammation, cellulitis, or subinvolution.

The solutions employed were of three different grades :

SOLUTION NO. I.

℞. Argent. nitratis (cryst.)........................ ʒ ss,

Aq. distil.................................. f. ℥ j. M.

This is the form to be applied to the interior of the cervical canal, which is always in a state of catarrhal inflammation, arising from the laceration or fissure which penetrates it. The remedy is applied down to the os internum, by means of a silver or whalebone probe wrapped with absorbent cotton.

SOLUTION NO. II.

℞. Argent. nitratis (cryst.)......................℈ iiss,

Aq. destil...............................f. ℥ j. M.

This solution is to be applied, or rather freely painted, over the external surface of the os and cervix uteri, by means of a large camel's hair brush, including the cavity of the laceration and granulating surface, until the whole presents the appearance of a grayish-white coating. The application usually gives no pain, but rather acts as a soothing or sedative agent, protecting the exposed sensitive branches of the torn nerves and the tender granulations. The coating will often remain from one to three days. While the remedy acts as a protective, it also acts as a mild but decided stimulant, to develop a new and more vigorous growth and a more healthy action. Thus, underneath this coating of silver, the processes of healing and filling up the gaping wounds, and the formation of a healthy cicatrix are in active operation.

At this stage, the remarkable action of the remedy is manifested in the reduction of engorgement, in stimulating the process of absorption, and in removing all hyperplastic deposit and hypertropy. By changing the morbid action in the wounded part, and affording an impervious coat, it suspends the absorption of septic matter, and interrupts the action of the cause of cellulitis, which gradually subsides under its influence. The silver applied in this manner never acts as a caustic or destructive agent, but only as a stimulant to vital action.

SOLUTION NO. III.

℞. Argent. nitratis (cryst.)......................ʒ ss,

Aq. destil...............................i. ℥ j. M.

Solution No. 3 is not only designed to be applied to the external surface of the cervix uteri, after the healing and closure of the laceration have been completed, but also when there remains a degree of hypertrophy or hyperplasia, or ordinary engorgement, accompanied with induration. A solution of this grade will generally reduce these conditions and leave the cervix soft and healthy.

It is unsafe to leave the cervix either in a hypertrophied or engorged state, as that might be the basis of future inflammation and reopening of the wound. A majority of the cases thus treated have since given birth to children. Some have borne three children since treatment, without difficulty. All have been entirely relieved of all unpleasant symptoms. Examinations held after the birth of each child showed a perfectly healthy os and cervix.

The application should be made every four days. During the intervals a douche of warm water, for the purpose of removing any accumulation of secretion, should be resorted to every morning.

It is the custom of the writer, in all cases of labor under his charge, to make a digital examination, at the time of the delivery of the secundines, of the os uteri, with the design of ascertaining the presence of laceration. If it exist, warm water douches, containing biborate of soda, boracic acid, and carbolic acid in solution, are used after the first day, with the intention of maintaining a perfect state of antisepsis, thus preventing infection, and promoting the healing of the wound by first intention, if possible. If fever should come on, notwithstanding this procedure, where no laceration can be detected, it would indicate a concealed fissure deep down in the cervical canal, near the internal os, which has never been reached by the antiseptic douches. In the event that healing by first intention fails to occur, antiseptic treatment and cleanliness of the wound are continued, and after two months the local treatment is commenced with the graduated solutions of nitrate of silver, with the certainty of a speedy restoration.—*College and Clinical Record.*

### CAUSE AND TREATMENT OF INFANTILE ECZEMA.

"Observations on the Cause and Treatment of Infantile Eczema and Allied Eruptions," was the subject of a suggestive paper read by Henry T. Byford, M. D.

He said, in the winter of 1880 he had been called to attend Mrs. R—— in her fifth confinement. Two of her other children were dead, and two more, apparently healthy at birth, had died in convulsions before they had completed one year of life. Both of the last two had suffered from scabby eruptions and had become somewhat emaciated, but had not been considered syphilitic. The child born at this time, seemingly healthy at first, soon broke out with what appeared to be an ordinary *eczema-pustulosom.* This eruption occurred on the scalp and on different parts of the body. The child suffered from progressive emaciation, and at the age of three weeks it would easily have been mistaken for a case of struma, to be treated with cod-liver oil. The patient was quickly relieved by calomel in minute doses and mercurial inunctions.

The next case referred to was that of Augustus E——, seen first in the summer of 1876. The child had pustular eczema of the scalp; great nocturnal restlessness, and suffered from progressive emaciation. There was no cause to suspect syphilis from the appearance of the child, but the father acknowledged to what seemed to be a perfectly cured attack of syphilis. The eczema rapidly disappeared, and the infant gained in flesh upon calomel powders and mercurial inunctions.

In contrast with these syphilitic cases, he next mentioned the case of E. H——, a child one year of age, fat and well nourished, with no possibility of a syphilitic taint, but suffering from an eczema of the head, which spread over the body in patches. Prolonged local treatment had not improved it. The disease soon disappeared under the use of one-fifth grain powders of calomel, given twice a day, and an ointment composed of a drachm of carbolic acid in an ounce of oxide of zinc ointment. The child had been over-fed, and was put upon a restricted diet. There was no return of the disease.

The next case was one in which the eruption invaded the eyelids, and caused great conjunctival sensitiveness. By the use of quarter-grain doses of calomel, given twice a day until a laxative effect was produced, then once a day, combined with the external application of carbolized oxide of zinc ointment, and a borax eye-water, the condition of the patient was soon greatly improved.

He succeded in relieving many other severe cases of eczema by the use of calomel, and it made no difference in what part of the body the eruption occurred or what the condition of the patient was otherwise.

That eczema so frequently occurs in infancy, the large size and great activity of the liver in early life, and the striking action of calomel, had led the author to associate indigestion with infantile eczema as cause and effect. In syphilitic cases the alterative powders often produced an amelioration in the skin trouble sooner than they could through their direct action upon the blood-poison. In all cases it is important to regulate the diet.

He knew of no authority who had considered derangements of the liver, and its accompanying digestive disorders, as the chief cause of eruptions of the skin, or who had recommended calomel as the chief remedy for its cure.

As calomel produced such prompt relief, and as improvement of digestion usually followed rather than attended the action of the remedy, one is led to believe that the cure is brought about not merely by improving digestion but by a removal of waste, irritating, matter from the system, and from the great efficiency of mercury over other laxatives. He believed that the irritating materials are not only in the retained fecal matter but also in the blood—the products of imperfect digestion, assimilation and excretion.

He was led to the use of calomel in eczema from noting its good effect in cases due to syphilis, and as these patients improved so well under its use, he next tried it in cases where syphilis was only suspected, and then in all cases.

The usual dose was from one-quarter to one-eighth of a grain, powder, given twice a day, the dose to be reduced if too great irritation of the bowels was produced. In cases of children over two and a half years of age, in order to avoid salivation, he usually gave purgative doses of calomel every six or eight days, and trusted to diet and other remedies in the meantime.

Dr. E. J. Doering said he had not used calomel in eczema, but had had good results from the use of corrosive sublimate. He intends to use calomel, after this, in such cases, and he thought nothing better could be found to use in chronic cases.

Dr. J. Zeisler remarked that he was very much interested in this paper, and, especially, to hear that calomel was useful in eczematous skin diseases. He had always held the view advocated by Hebra, that eczema was caused by external causes and required external applications for its cure. Calomel might be of use in cases due to syphilis. He would not use blue ointment on young children. He asked whether it would not be best to use some external application in cases where there were thick masses of crusts covering the diseased part, and also whether the author of the paper had found salicylic acid useful in this disease.

Dr. C. W. Purdy thought retained excreta a cause of eczema, and called attention to the close relation existing between the intestinal tract and the skin, the condition of one affecting the other.

He had seen cases cured by the use of calomel and would favor this treatment.

Dr. J. A. Robison said that Dr. Byford had found mercury beneficial in syphilitic and non-syphilitic cases, probably because given in small doses it was a general tonic, as iron or cod-liver oil, increasing the number of red blood corpuscles and enriching the blood. Thus it counteracted any dyscrasia present.

Dr. Byford concluded by saying that Hebra was doubtless right in saying that eczema is caused often by external causes, but in some cases it was clear to him that the disease is due to causes arising within the system itself. In later years Hebra had modified his ideas of this disease somewhat. Although eczema could be cured by external applications, when so treated it is very apt to return. It is not so when removed by the use of calomel. External medication would often hasten the cure. He did not use salicylic acid. He believed that calomel in these cases acted as an agent causing eliminations as well as being a tonic.—*Chicago Medical Journal and Examiner.*

